# Quantifying yield behaviour in metals by X-ray nanotomography

**DOI:** 10.1038/srep34346

**Published:** 2016-10-04

**Authors:** M. Mostafavi, R. Bradley, D. E. J. Armstrong, T. J. Marrow

**Affiliations:** 1 Department of Mechanical Engineering, Queen's Building, University of Bristol, University Walk, Bristol BS8 1TR, UK; 2 Manchester X-ray Imaging Facility, Photon Science Institute, Alan Turing Building, The University of Manchester, Manchester M13 9PL, UK; 3 Department of Materials, University of Oxford, Parks Road, Oxford, OX1 3PH, Oxford, UK; 4 Oxford Martin School, University of Oxford, 34 Broad Street, Oxford, OX1 3BD, UK

## Abstract

Nanoindentation of engineering materials is commonly used to study, at small length scales, the continuum mechanical properties of elastic modulus and yield strength. However, it is difficult to measure strain hardening via nanoindentation. Strain hardening, which describes the increase in strength with plastic deformation, affects fracture toughness and ductility, and is an important engineering material property. The problem is that the load-displacement data of a single nanoindentation do not provide a unique solution for the material’s plastic properties, which can be described by its stress-strain behaviour. Three-dimensional mapping of the displacement field beneath the indentation provides additional information that can overcome this difficulty. We have applied digital volume correlation of X-ray nano-tomographs of a nanoindentation to measure the sub-surface displacement field and so obtain the plastic properties of a nano-structured oxide dispersion strengthened steel. This steel has potential applications in advanced nuclear energy systems, and this novel method could characterise samples where proton irradiation of the surface simulates the effects of fast neutron damage, since facilities do not yet exist that can replicate this damage in bulk materials.

Engineering design requires knowledge of the continuum mechanical properties of materials, which include the elastic modulus and the plastic properties of yield stress, ultimate strength, and tensile ductility. In bulk materials, these are measured by standardised procedures such as ASTM E8^1^ to ensure the data are representative and accurate. However, materials testing at this length scale is not always possible or appropriate, and smaller scale test methods are required, particularly for the materials of advanced nuclear energy systems.

The extreme conditions of neutron irradiation in future nuclear fission and fusion power plants can damage their structural materials^2^,^3^. This damage occurs as the kinetic energy of fast neutrons is absorbed through successive interactions with atomic nuclei; in turn, disturbed atoms displace their neighbours in a cascade of damage. Most of the atomic displacements are transitory, but the residual defects in the crystal lattice can affect its properties, particularly the behaviour of the dislocations that enable yield by plastic deformation. The strain hardening ability, which quantifies the increase in yield strength with increasing strain by the multiplication of dislocations, may be changed by irradiation-induced defects as they affect dislocation motion and interactions. The ferritic structural steels of the current generation of nuclear fission plant lose ductility with neutron irradiation, with significant consequences for engineering design. This was originally considered to be due to a reduction in the rate of strain hardening^4^, but the embrittlement of these materials is known now to be caused by the irradiation-induced increase of yield stress, which reduces the strain required to achieve the critical stress for plastic instability^5^. Hence, there is actually no significant effect of irradiation on strain hardening in these steels, but quite detailed studies of irradiated material properties and deformation were required to achieve this understanding. Similar studies are now needed to design and select the new structural materials of future fission and fusion energy systems, but the facilities that can replicate or accelerate the necessary irradiation spectra, neutron dose, dose rates and temperatures do not yet exist^6^. Some irradiation effects may be simulated using highly accelerated charged particles (ions), but these affect only a surface layer that is up to a few tens of micrometres deep, at most. This is sufficient to study defect structures and dislocation behaviour^7^,^8^, but the effects of ion irradiation on mechanical properties must be studied by micromechanical testing^9^,^10^,^11^.

Micromechanical tests do not necessarily measure continuum material behaviour due to the granular nature of crystalline materials, but with appropriate consideration of the length scales, the data may be used in the predictive models of continuum behaviour^12^ that are needed to support the efficient design of new steel alloys; for example see Zinkle’s & Ghoniem’s work^13^. The elastic modulus can be measured by nanoindentation^14^, with crystal anisotropy studied with suitably oriented micro-machined beams^15^. Plastic behaviour is more challenging because although micromechanical tests can provide data on dislocation mobility and the onset of yield^16^, the strain hardening is quite difficult to study. The underlying micromechanical factors, such as the gradients of dislocation density that govern the microplastic behaviour of materials have been measured through micro-analysis methods such as Laue diffraction^17^,^18^,^19^,^20^,^21^, but whilst such measurements significantly aid understanding of deformation mechanisms, they do not provide data that can be used in engineering design^22^.

In principal, the plastic properties of yield and strain hardening may be extracted from a detailed analysis of a hardness indentation test since the deformation that occurs under an indenter of known geometry with precise measurement of the load (*P*) in response to a known applied displacement (*h*) is well defined^23^. Three-dimensional finite element (FE) simulations for conical, Vickers, Berkovich and spherical indentations of ductile materials all demonstrate sensitivity of the deformation field to the strain hardening exponent and yield stress^24^,^25^,^26^,^27^, and plastic properties have been extracted using instrumented indentation tests^10^,^28^,^29^. The obtained properties can have significant uncertainty due to a lack of knowledge of experimental factors that include indenter alignment, surface friction and the contact area between the indenter and the material^30^, although these effects may be ameliorated by using multiple^10^,^31^ and spherical indentations^32^. The extraction of material properties from indentations is aided by the acquisition of extra information. For instance, surface characterisation by Atomic Force Microscopy (AFM) has been applied to measure the indentation contact area more accurately, improving the reliability of the elastic property measurement^33^,^34^.

The significant problem is that the measured load-displacement data for a single indentation do not provide a unique solution of the elastic modulus, yield stress and strain hardening properties^24^,^25^,^35^, and whilst micro-pillar compression tests^11^ have been used to characterise yield and strain hardening behaviour, the deformation within the pillar geometry can be non-uniform. To improve the accuracy of micromechanical test analyses, there is a need for better information on the distribution of plastic deformation.

The deformation of the material that is beneath an indentation can be observed and measured as a three-dimensional displacement field via digital volume correlation of X-ray computed tomographs^36^,^37^. With this additional information, optimised fitting of FE solutions to the measured data can extract the material properties with greater confidence^36^. Here we apply this methodology at the nanoscale to measure strain hardening from a single indentation in a micro-mechanical test specimen of oxide dispersion strengthened (ODS) steel; this class of materials is a candidate for structural components in fusion and Generation IV nuclear fission power plant^38^.

## Results

Plansee’s PM2000 grain class 4 (DIN - CrAl 216) Oxide Dispersed Strengthened steel^39^ was used in this study. Its composition (weight %) is 19 Cr, 5.5 Al, 0.5 Ti and 0.5 Y_2_O_3_ (balance Fe). At room temperature, the standard tensile properties of the bulk material are Young’s Modulus *E* = 190 GPa, yield stress at 0.2% proof strain *σ*_*y*_ = 1110 *MPa*, tensile strength (TS) *σ*_*TS*_ = 1185 *MPa* and 14.0% elongation at rupture^40^. These parameters give an equivalent Ramberg-Osgood strain hardening exponent, *n* = 0.026^41^. The grain size is around 1 μm and it contains a distribution of yttrium oxide particles of ~20 nm diameter^42^.

The specimen (~1 mm × ~1 mm × ~8 mm) was prepared from an as-received PM2000 plate using a slow speed diamond saw. One end was ground with silicon carbide abrasive paper (~2 μm particle size) to a point, onto which a pillar was micro-machined with an annular milling pattern of 30 keV Gallium ions in a Zeiss Auriga FIB-SEM (focussed ion beam-scanning electron microscope). A high beam current (16 nA) milled a 30 μm diameter pillar, which was thinned to a final diameter of 16 μm with progressively lower beam currents to a final polishing step at 200 pA. A fiducial cross was marked on the pillar side using a 50 pA beam current to aid registering of tomography datasets.

X-ray nanotomography was carried out using a Zeiss Xradia 810 Ultra system, which is a Fresnel zone plate X-ray microscope, with phase contrast obtained via the Zernike method^43^. Tomography scans were taken using 50 nm resolution optics at an exposure time of 225 seconds per radiograph at 8 keV beam energy, with 541 radiographs recorded as the sample was rotated over 180°. The specimen’s dimensions were chosen to optimise the X-ray transmission at the maximum energy of this instrument. The volumetric data were reconstructed with a voxel size of 32.4 nm using the filtered back projection algorithm via the TomoTools^44^ interface to the ASTRA reconstruction toolbox^45^.

The nanoindentation was performed on the pillar’s flat surface using a MicroMaterials NanoTest instrument with a diamond Berkovich tip. The indenter displacement applied was 1 μm at a loading rate of 20 nm/s, with a 2 second hold at the maximum displacement followed by unloading at 0.1 nm/s. An indentation depth of approximately 1 μm is sufficient to obtain a depth-insensitive hardness in this steel^46^, and was chosen also due to the restricted size of the specimen. Such an indentation would be suitable to study the properties of a proton irradiated material, but not for self-ion irradiation which has less penetration^47^.

**Figure 1:**
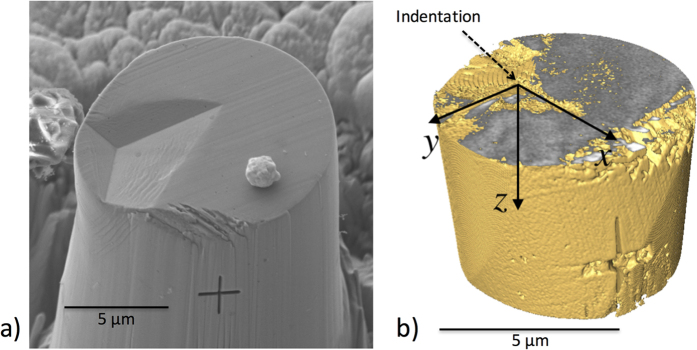
The pillar of PM2000 ODS steel after nanoindentation. (**a**) Scanning electron microscope image (a gold particle of ~1 μm diameter applied to the indented surface was used as a marker during the set-up of the tomograph); (**b**) surface rendering of tomographic data, which has been cropped just below the horizontal surface to remove image artefacts and to highlight the indentation as the impressed region. The coordinates are defined relative to the deepest point of the indentation.

**Figure 2:**
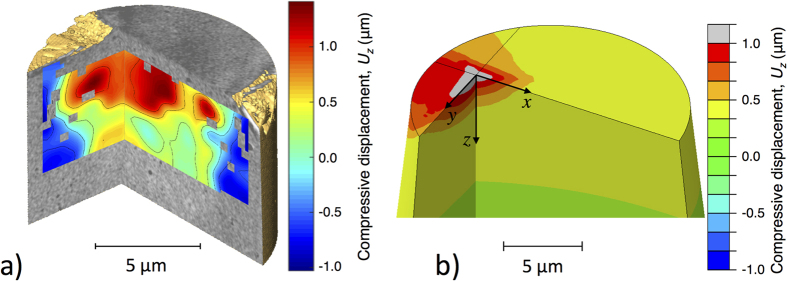
The displacement field under the indentation. (**a**) Measured by digital volume correlation of X-ray computed tomographs. The tomograph has been virtually sliced along orthogonal planes passing through the deepest point of indent to exhibit the contrast from distributions of yttrium-rich particles in the microstructure. Contours of the vertical (downwards) displacement (Uy) in those vertical planes, obtained from the 3D displacement field, are overlaid on the image; (**b**) an example of a finite element (FE) simulation of the nanoindentation experiment. The vertical (downwards) displacement (Uy) field is presented in the orthogonal planes that pass through the deepest point of the indentation.

A three-dimensional rendering of the tomographic data after indentation is shown in Fig. 1, compared with a scanning electron microscopy observation. A horizontal crop has been made in the tomograph to remove image-artefacts at the surface, on which the impression of the indentation can be observed. In Fig. 2, orthogonal slices section the visualisation, passing through the deepest point of the indentation. The *x*-axis of the coordinate system is aligned with the longer axis of the indentation impression. The steel’s fine microstructure has regions of variable X-ray attenuation, with fine highly attenuating (bright) yttrium-rich particles and less attenuating (dark) coarse Al_2_O_3_ and Ti carbide/nitride particles^48^,^49^ being quite clearly observed in Fig. 2. Digital Volume Correlation (DVC) is a full-field displacement measurement technique based on the tracking of patterns in images^50^. The choice of imaging method is important, as a sufficient variation in grey scale image intensity (i.e. “speckle”) is required for good correlation. In the present work, the heterogeneous distribution of the yttria-rich particles, which are not well resolved, provided sufficient speckle for the DVC measurement of the three-dimensional displacement field beneath the indentation.

DVC analysis considers the volumetric datasets in smaller ‘interrogation subsets’ and maps their displacement and deformation between successive images; it is a key tool in quantitative tomography^51^. The LaVision Davis 8.0 software was used, with a multiple step cross correlation process with a cubic 256 voxel subset (75% overlap and 2 pass) followed by 128 (75% overlap 3 passes) and finally 64 (75% overlap and 4 passes) voxel subsets. Analysis of tomographs between which the specimen was physically translated by 1 μm gave a standard deviation of the maximum displacement magnitude of 1.23 voxels (i.e. 39.4 nm), which is taken as the displacement measurement uncertainty.

The vertical component of the 3D displacement field is visualised in Fig. 2a, overlaid on a rendering of the indented sample tomograph. The displacements are relative to the original position of the material at the deepest point of the indentation, and describe the residual deformation after removal of the indentation. The tomographs were manually registered before the DVC analysis. Because of the off-centre position of the indentation, the pillar bent towards one side (see Fig. 1a), hence the measured displacement field is a combination of the rigid body translation, rigid body rotation, and deformation due to indentation. We have used an efficient technique based on an inverse Euler angle rotation^36^ to decompose (and present) the displacements due to indentation deformation independently from those caused by rigid body translation and rotation.

Three-dimensional elastic-plastic FE modelling of the indentation extracted the plastic properties of the steel from the experimental data by optimisation, following a method previously applied to a macroscopic Hertzian indentation in a ductile metal^36^. A three dimensional FE model of the pillar was created in Abaqus 6.14, using 47000 8-node brick elements with linear elastic – linear plastic (kinematic hardening) material properties; the elastic properties were Young’s modulus *E* = 190 GPa and Poisson’s ratio *ν* = 0.3. The Berkovich indenter was simulated using 960 rigid shell elements. Contact between the specimen and the indenter was frictionless, or with a coefficient of friction of 0.3. The boundary conditions of the simulation directly reproduced the experiment, using measurements of the pillar geometry, indenter alignment and position that were provided by the tomographs, and the indentation displacement after unloading that was obtained from the DVC analysis. The indenter tip was 6.14 μm from the pillar centre, with the indenter axis aligned 8.5° from vertical. The reaction force in the simulation matched the maximum applied indentation load. A three dimensional visualisation of the calculated vertical component (*U*_*z*_) of the displacement field is shown in Fig. 2b, with sections along the orthogonal *yz* and *xz* planes.

**Figure 3:**
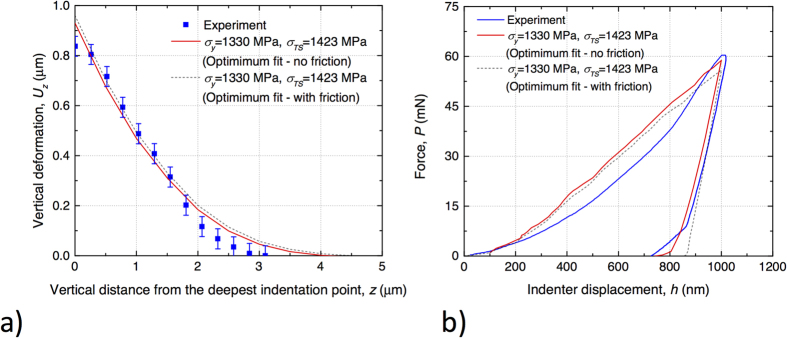
Nanoindentation data and results of finite element (FE) simulations of the nanoindentation experiment. (**a**) Measured and simulated relation between indentation load (P) and indenter displacement (h); (**b**) measured and simulated vertical compressive displacement (Uy) below the deepest point of the indentation, as a function of distance below the indent.

The largest displacements are the vertical components (*U*_*z*_) along a vertical path below the indentation, and these were used to optimise the material properties of the FE model. The displacement field, as a line-profile of the vertical displacements under the indenter tip (Fig. 3a) was compared with the experimental data. The *P-h* (load-displacement) data are shown in Fig. 3b. The FE simulation of the experiment was optimised by the exhaustive search method, using 85 simulations with frictionless contact of varying normalised yield stress *σ*_*y*_/*E* and linear strain hardening rate. For simplicity, this is represented as a ratio of tensile stress (*σ*_*TS*_) at 14% strain to the yield stress (*σ*_*y*_, at 0.2% strain). The *P-h* simulations with the same material properties are weakly sensitive to the coefficient of friction (i.e. *μ* = 0.3 between diamond and steel - Fig. 3b). The Berkovitch indenter included angle is 70.3°, and a significant friction effect on the normal force has only been measured on tips with included angles of 50° or less^30^. Friction also has a negligible effect (less that 3%) on the simulated displacement field (Fig. 3a).

**Figure 4:**
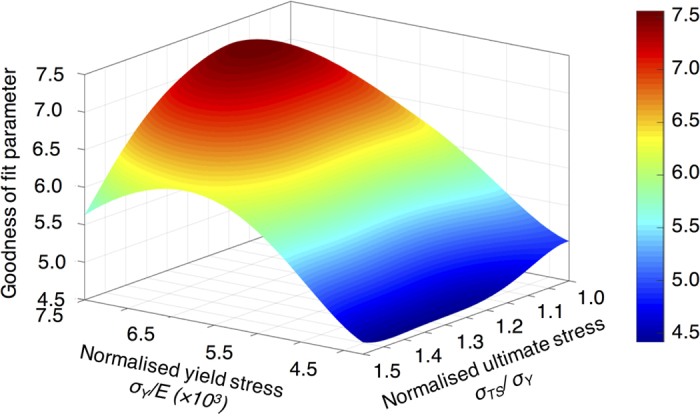
Goodness of fit parameter (see Eq. 1); maximum experiment-model correlation was observed at 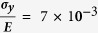 and 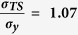.

For each simulation the normalised square root difference (i.e. residual) between the vertical component of the displacement below the indent between FE and experiment was calculated. The summation of the compressive experimental displacement below the indent was normalised by the residue to calculate the goodness of fit:


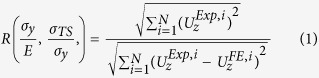


where *U*_*z*_ denotes the vertical component of the displacement vector. 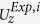 is the experimentally measured 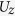 at the *i*th displacement vector measured below the indent and *N* was selected to be 8 (i.e. data were used up to 4 μm below the deepest point of the indent). 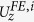 are the respective FE displacements. The maximum goodness of fit (Fig. 4) provides optimal fitting material properties at 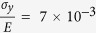 (i.e. 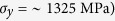 and 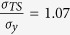.

Physically, it is the stress-strain curve after yield that defines the material strain hardening behaviour. This can be simulated by a number of models, each with its own fitting parameters. The most widely used in engineering is a Ramberg-Osgood model (deformation plasticity) as the strain hardening behaviour can be described by one main parameter: the hardening exponent. However, the deformation plasticity model implemented in most finite element simulation software is a nonlinear elastic model that upon unloading, leaves no permanent plastic deformation. This is unsuitable for simulating the indentation experiment, as it was observed *ex-situ* with the deformation measured after unloading. The FE simulations, therefore, exploited kinematic hardening to describe plastic deformation after unloading. The equivalent Ramberg-Osgood strain hardening exponent^41^ obtained from the analysis of the indentation is 0.027, which compares well with the expected hardening exponent of 0.026 for Class 4 PM2000^40^.

The obtained yield stress is higher than the standard properties of this steel grade^40^, but is very close to the yield stress calculated by a conventional nano-indentation elastic-plastic analysis of the *P-h* data^52^ (i.e. 1325 MPa). A conventional elastic-plastic analysis of indentation data^52^ obtains a very low strain hardening exponent, *n* = 0.003, when applied to these data, which may be attributed to an inaccurate assumption of the sub-indentation strain field. The difference between the obtained and macroscopic yield stress is likely to be caused by material heterogeneity; the yield strength of PM2000 is grain size dependent^53^ and the volume of material affected by the nanoindentation is significant relative to the grain size. Tensile data reported in other studies of bulk specimens of PM2000 steels^53^,^54^ provide strain hardening exponents between 0.04 and 0.17.

As the material in this study had micrometre size grains and the examined volume was approximately 800 μm^3^, we chose a continuum model (i.e. kinematic hardening) as being most appropriate. Alternative continuum models that provide the equivalent stress-strain curve could also be used. If a similar study were to be performed in a very coarse grained specimen (e.g. representative of single crystals), then more representative models such as gradient plasticity^55^ or discrete dislocation-plasticity^56^ would be appropriate, and for deformation in a limited number of grains, a crystal plasticity model could be used^57^.

The method of exhaustive search was used in the current study due to its simplicity and the limited number of parameters that were to be fitted (i.e. yield stress and the ratio of tensile strength to yield stress). With a greater number of fitting parameters, such as those required for other models, the more time-consuming, yet more accurate identification processes, such as finite element model updating (FEMU)^58^ or the virtual field method (VFM)^59^, could have been used.

## Conclusion

This analysis demonstrates the novel use of full field displacement data to characterise a nanoindentation and so obtain the strain hardening behaviour. It could be applied to examine the sensitivity of the strain hardening, or strain softening, behaviour of structural steels to ion-irradiation in order to investigate the potential effects of high doses of fast neutron irradiation; care should be taken however, that the irradiation damaged zone has sufficient depth and uniformity of damage to fully accommodate the indentation plastic zone. The FE optimisation to characterise the yield stress and strain hardening currently uses only one displacement component along a linear path. A straightforward exhaustive search has been used but more advanced optimisation algorithms would reduce the computation time and allow more data, such as the full 3D displacement field, as input. The current analysis used only observations before and after indention. *In situ* observations obtained during indentation, with the microstructure under load and potentially at elevated temperature, could provide greater insight and progressive observations that would permit analysis of non-linear strain hardening or softening behaviour.

## Additional Information

**How to cite this article**: Mostafavi, M. *et al*. Quantifying yield behaviour in metals by X-ray nanotomography. *Sci. Rep.* **6**, 34346; doi: 10.1038/srep34346 (2016).
